# Stem cell mechanical behaviour modelling: substrate’s curvature influence during adhesion

**DOI:** 10.1007/s10237-017-0888-4

**Published:** 2017-02-21

**Authors:** M. Vassaux, J. L. Milan

**Affiliations:** 1 Institute of Movement Sciences, Aix Marseille University, CNRS, Marseille, France; 2 Department of Orthopaedics and Traumatology, Institute for Locomotion, APHM, Sainte-Marguerite Hospital, 13009 Marseille, France

**Keywords:** Stem cell adhesion, Numerical mechanical analysis, Substrate curvature, Intracellular mechanosensitivity

## Abstract

Recent experiments hint that adherent cells are sensitive to their substrate curvature. It is already well known that cells behaviour can be regulated by the mechanical properties of their environment. However, no mechanisms have been established regarding the influence of cell-scale curvature of the substrate. Using a numerical cell model, based on tensegrity structures theory and the non-smooth contact dynamics method, we propose to investigate the mechanical state of adherent cells on concave and convex hemispheres. Our mechanical cell model features a geometrical description of intracellular components, including the cell membrane, the focal adhesions, the cytoskeleton filament networks, the stress fibres, the microtubules, the nucleus membrane and the nucleoskeleton. The cell model has enabled us to analyse the evolution of the mechanical behaviour of intracellular components with varying curvature radii and with the removal of part of these components. We have observed the influence of the convexity of the substrate on the cell shape, the cytoskeletal force networks as well as on the nucleus strains. The more convex the substrate, the more tensed the stress fibres and the cell membrane, the more compressed the cytosol and the microtubules, leading to a stiffer cell. Furthermore, the more concave the substrate, the more stable and rounder the nucleus. These findings achieved using a verified virtual testing methodology, in particular regarding the nucleus stability, might be of significant importance with respect to the division and differentiation of mesenchymal stem cells. These results can also bring some hindsights on cell migration on curved substrates.

## Introduction

Life and fate of mesenchymal stem cells (MSCs) are dependent on perceived mechanical cues: migration, adhesion, differentiation or even apoptosis can be controlled by adapting stresses or strains sensed by the cell (Li et al. 2002; Bershadsky et al. 2003; Vogel and Sheetz 2009). The environment surrounding the cell plays a significant role in the regulation of these mechanical cues (Vogel and Sheetz 2006); a well-known example is the sensitivity of cells migration to substrate stiffness called durotaxis (Trichet et al. 2012).

For example, the long-term adhesion of bone cells has been demonstrated to be statistically better correlated with the roughness organization of topography than with any other properties of the substrate (Anselme and Bigerelle 2005). Indeed, the morphology of the cell environment influences the cell shape, and it has been shown that cells shape controls cells fate (Folch and Toner 2000; Tan et al. 2003); therefore, morphology of the environment has chances to influence cell fate.

The investigation of cell responses to diverse topographical cues has been intensely performed with planar or anisotropic topographies (Chen et al. 1997; Théry et al. 2005; Park et al. 2009; Lee and Yang 2012; Kim et al. 2012). However, studies on smooth 3D, cell-scale topographies are more rare, even though more representative of biological structures (Nagasawa 2006; Shechter et al. 2013), inducing specific cell responses (Bigerelle et al. 2011). Only recently, the use of electro-erosion (Bigerelle et al. 2002) or lithography (Mathur et al. 2012; Song et al. 2015), for example, has enabled the manufacturing of such smooth topographies. The development of these techniques helped to study adherent cells behaviour dependence to curvature and to provide associated macroscopic mechanical data, such as shape or traction forces (Soiné et al. 2016).

Coupled approach based on experiments and simulations (Kim et al. 2012) has demonstrated that the cell migration on curved surfaces is in part driven by haptotaxis: concave curvatures basically show higher probabilities of creating ligand–receptor bonds promoting migration. Nevertheless, unclear mechanisms related to actin polymerization and actomyosin contractility, allowing cells probing of the curvature, cannot be excluded (Song et al. 2015). Thus, the mechanisms that rules the global behaviour and sensitivity of single cells on curved topographies remain unclear and have to be explored.

Models have also been used to investigate mechanical stresses in adherent cells on curved topographies (Sanz-Herrera et al. 2009). In the present paper, similarly, we propose to investigate internal mechanical state of single adherent MSC on a various range of 3D spherical curvatures. We want to discriminate substrates topography influence on the stresses and strains of intracellular structures, such as the cell membrane, the stress fibres and with further emphasis on the nucleus. We believe that these results can find significance in cell decisions during migration in favour of latter mitosis and differentiation or in favour of enhanced motility.

Investigation of the intracellular mechanical behaviour, often limited in experiments, has been achieved by means of computational modelling. One of the theories on how cells generate and transmit forces relies on the description of cytoskeleton as a tensegrity structure (Chicurel et al. 1998; Wang et al. 2001). The microtubules are considered to be the compression component and other filaments the tension components. Computational models based on the tensegrity structures theory have been efficient to reproduce the mechanical phenomena observed in adherent cells: strain-hardening (Wang et al. 1993), contractility and induced stiffening (Wang et al. 2002; Pourati et al. 1998; Wendling et al. 2003; Wang and Sun 2012) and visco-elastic properties (Cañadas et al. 2002; Sultan et al. 2004).

Despite the various range of computational models of cell mechanics available (Lim et al. 2006), the aforementioned qualities of models inspired of tensegrity structures motivated our choice to model the cell as two encapsulated membranes in between which the cytoskeleton is explicitly described (Ujihara et al. 2010; Milan et al. 2007, 2013, 2016). In addition, such discrete modelling provides us direct access to the mechanical state of the different intracellular structures that we want to analyse.

The cell numerical model is later shown to reproduce appropriately certain mechanics of adherent MSC. We make use of this numerical tool to investigate specifically the strains and forces in the microtubules, the actin-based constituents of the CSK, the nucleus membrane, the cell membrane and the nucleoskeleton. All of them are studied while varying the curvature of the substrate on which the cell are made adhering.

## Methods

### Computational method for cell mechanics modelling

Single cell modelling is achieved using non-smooth contact dynamics (NSCD) (Jean 1999), a discrete element-based method, by means of the LMGC90 software (Dubois 2006). Previous models of single cell mechanics based on NSCD can be found in (Milan et al. 2007, 2013, 2016). This method is based on non-elastic body dynamics, and the structure modelled is discretized in a set of spherical particles, each particle *i* verifying the equilibrium Eq. (1).

1 $$\begin{aligned} {\underline{F}}_{i} + \sum \limits ^{N}_{j=1} {\underline{F}}_{i,j} = m_i {\underline{a}}_{i} \end{aligned}$$

where ${\underline{F}}_{i}, {\underline{a}}_{i}$ and $m_i$ are, respectively, the external force (boundary condition) applied to, the acceleration of, and the mass of the particle *i* and ${\underline{F}}_{i,j}$ is the interaction force between particles *i* and *j*, *j* being one of the *N* particles interacting with *i*, among all the particles constituting the discretized structure.

Particles interact in several manners in order to reproduce interactions found inside the cell. Three types of interactions are required: compressive contact, tensile cable and spring. Such mechanical description of the cell is equivalent to tensegrity structures (Fuller 1961), namely an assembly traction and compression bearing elements. Such modelling approach is particularly suited for discrete structures (e.g. filaments networks); however, more continuous structures (e.g. membranes) are intricate to simulate with a rigid-bodies model. Indeed, to preserve impermeability of the membranes (e.g. avoid cytosol flowing out the cell) while allowing their deformation, a high number of particles in the membrane are required which lead to pay high computational costs compared to continuum-based modelling techniques (e.g. finite elements method).

The tensegrity organization of the cytoskeleton structure is originally inspired from the work of (Maniotis et al. 1997). The three interactions are briefly defined.

#### Contact

**Fig. 1:**
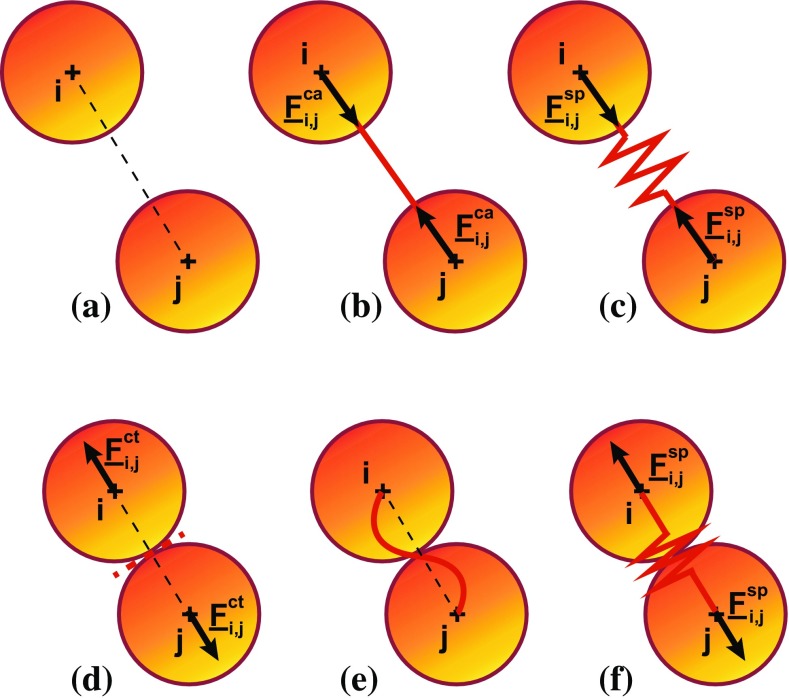
Schematic drawing of the three interactions between particles: **a**–**d** contact inducing compression force when the particles touch, **b**–**e** cable inducing tension force when longer than its initial length and **c**–**f** spring inducing compression or tension force when, respectively, shorter or longer than its initial length

Contact interaction (Fig. 1a–d) allows particles to repel each other. Contact is defined in the strict sense of Signorini’s contact problem (2), which resolution is made possible by the NSCD implicit solution algorithm. Frictionless contact is considered here.

2 $$\begin{aligned}&F^{\mathrm{ct}}_{i,j} \ge 0; \quad g_{i,j} - \left( r_i + r_j\right) \ge 0; \nonumber \\&\qquad F^{\mathrm{ct}}_{i,j}.\left( g_{i,j} - \left( r_i + r_j\right) \right) = 0 \end{aligned}$$

where $F^{\mathrm{ct}}_{i,j}$ is the magnitude of the contact force applied by the particle *j* on *i*; $g_{i,j}(=|{\underline{x}}_{i}-{\underline{x}}_{j}|_2$) is the gap between centroids of the particles *i* and *j*, with ${\underline{x}}$ the position vector; and $r_i$ and $r_j$ are the radii of the particles *i* and *j*.

#### Cable

Cable interaction (Fig. 1b–e) enables two particles to solely pull on each other (3), when the distance between the two particles exceeds the length of the cable.

3 $$\begin{aligned} F^{\mathrm{ca}}_{i,j} = \left\langle k_{i,j}\left( \frac{g_{i,j}-g^0_{i,j}}{g^0_{i,j}} + \tau _{i,j}\right) \right\rangle _+ \end{aligned}$$

where $F^{\mathrm{ca}}_{i,j}$ is the magnitude of the traction force applied by the particle *j* on *i*, and $k_{i,j}$, $g^0_{i,j}$ and $\tau _{i,j}$ are, respectively, the stiffness defined as force per strain, namely the product of the cross section with the Young’s modulus, the initial length and the pre-strain of the cable linking the particles *i* and *j*; the brackets $\langle . \rangle _+$ refer to the positive part of the scalar in between. A positive pre-strain leads to a positive offset of the global strain, which induces a nonzero tension force in the cable at zero strain. Inversely, a negative pre-strain delays the generation of a tension force in the cable when stretched.

#### Spring

Spring interaction (Fig 1c–f) enables two particles to pull as well as to push on each other (4).

4 $$\begin{aligned} F^{\mathrm{sp}}_{i,j} = k_{i,j}\left( \frac{g_{i,j}-g^0_{i,j}}{g^0_{i,j}} + \tau _{i,j}\right) \end{aligned}$$

where $F^{\mathrm{sp}}_{i,j}$ is the magnitude of the force applied by the particle *j* on *i*, and $k_{i,j}, g^0_{i,j}$ and $\tau _{i,j}$ are, respectively, the stiffness defined as force per strain, the initial length and the pre-strain of the cable linking the particles *i* and *j*.

### Implementation of the cell non-elastic body mechanics model

Intracellular components are modelled by means of a set of rigid particles interacting by means of one of the aforementioned constitutive laws.

**Fig. 2:**
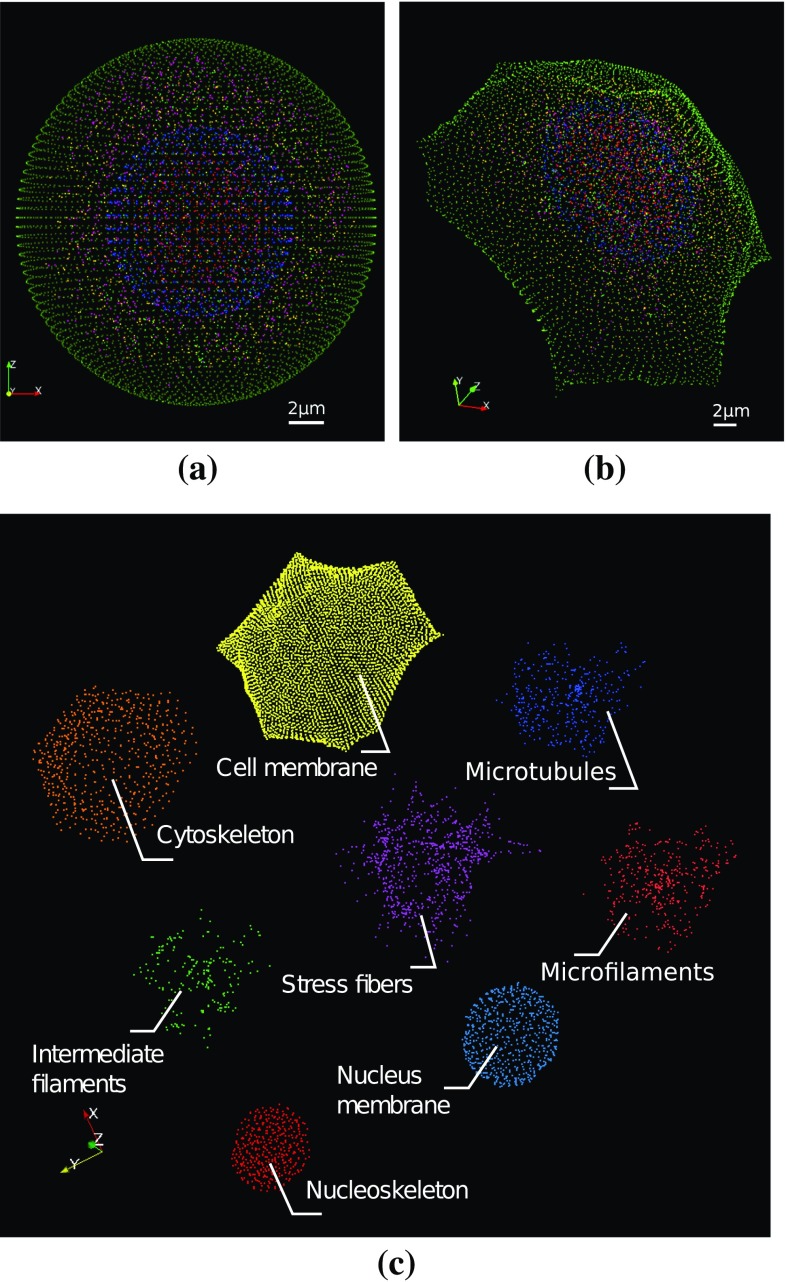
Visualization of the cell model in its **a** initial spherical and **b** final spread configurations. Spatial distribution of the particles centroids constituting the intracellular structures in the spread configuration (**c**), each particle *colour* refers to a specific intracellular structure

In its initial configuration, the cell is generated as a spherical structure (Fig. 2a). The generation of the cell in its initial state is not entirely deterministic, and some components of the cytoskeleton (CSK) are generated probabilistically (intermediate filaments, microfilaments, microtubules and stress fibres), in order to introduce some anisotropy in the cell structure. In the probabilistically defined set of particles, the position of a particle *i* is described using a spherical coordinate system, which origin is the nucleus centre. The variability is therefore imposed on the radius $r_i$, and the two angles $\theta _i$ and $\phi _i$.

Depending on the tested cell configuration, some or all of the following intracellular constituents will be included in the mechanical model (Fig. 2c).

#### Nucleoskeleton

The nucleoskeleton (NS) is modelled as a dense spherical packing of particles interacting solely through contact. Optimal density of the packing is first ensured to avoid possible compressibility of the packing during simulations. In that way, the NS is approximated as a fluid in the context of particular methods. Particles of the NS interact as well through contact with particles of the nucleus membrane.

#### Nucleus membrane

The nucleus membrane (NM) is considered as an impermeable membrane allowing the NS to deform while conserving its volume. It is modelled as an hollow sphere made of one layer of initially overlapping particles. Particles overlap initially in order to allow an extension of the surface of the membrane without loss of impermeability. Particles of the membrane interact with each other solely through cables of stiffness $k_{\mathrm{NM}}$. A particle of the set is assumed to interact with other particles of the set located at an initial distance lower than $l_{\mathrm{NM}}$. Particles of the NM interact as well through contact with particles constituting the NS, the cytosol and the CSK.

#### Cell membrane

Identically to the NM, the cell membrane (CM) is considered as an impermeable membrane. Thus, it is also modelled as an hollow sphere made of one layer of initially overlapping particle. However, this time, particles of the membrane interact with each other through pre-strained cables, due to the homogenization in the membrane of the actomyosin cortex. CM cables have a stiffness $k_{\mathrm{CM}}$ and a positive pre-strain $\tau _{\mathrm{CM}}$. The positive pre-strain induces an initial tension force in the CM, which reproduces its contractile behaviour. Particles of the CM interact as well with other particles of the set located at an initial distance lower than $l_{\mathrm{CM}}$. Particles of the NM interact as well through contact with particles constituting the cytosol and the CSK.

#### Cytosol

The cytosol (CS) is identically defined as the NS. The CS is modelled as a dense spherical packing of particles, outside from the NM, filling the CM. Particles of the CS interact through contact with each other, as well as with particles constituting the NM, the CM and the CSK.

#### Intermediate filaments

Intermediate filaments (IFs) are considered as a network of interconnected loose cables surrounding the NM. The network of IF is modelled as a sparse set of particles interacting with each other through cables. A particle of the set is assumed to interact with other particles of the set or with particles of the NM (Green et al. 1986) located at an initial distance lower than $l_{\mathrm{IF}}$. The looseness of the IF is reproduced by means of a negative pre-strain $\tau _{\mathrm{IF}}$. The IFs are assumed to be preferentially located nearby the nucleus; therefore, the distance of the particle *i* from the nucleus centre is drawn in a normal (${\mathscr {N}}$) distribution, with a rather small specific variance $\sigma ^2_{\mathrm{IF}}=\frac{r_{\mathrm{CM}}}{8}$. However, the two positioning angles of particle *i* are drawn from uniform (${\mathscr {U}}$) distributions (5).

5 $$\begin{aligned} \left\{ \begin{array}{l l} r_i={\mathscr {N}}\left( \mu =r_{\mathrm{NM}}, \sigma ^2=\sigma ^2_{\mathrm{IF}} \right) ; \quad r_{\mathrm{NM}}< r_i < r_{\mathrm{CM}}\\ \theta _i={\mathscr {U}}\left( 0,2\pi \right) \\ \phi _i= {\mathscr {U}}\left( -\pi ,\pi \right) \end{array} \right. \end{aligned}$$

where $r_{\mathrm{NM}}$ and $r_{\mathrm{CM}}$ are, respectively, the radii of the NM and of the CM and $\sigma ^2_{\mathrm{IF}}$ is the specific variance attributed to the distribution of particles of the IF network.

#### Microfilaments

Similarly to the IF, the microfilaments (MFs) of actin are considered as a network of interconnected cables. A particle of the set is assumed to interact with other particles of the set located at an initial distance lower than $l_{\mathrm{MF}}$. However, MF cables, unlike IF cables, are not loose but initially contracted by the action of myosin, which is reproduced by means of a positive pre-strain $\tau _{\mathrm{MF}}$, similarly to the CM. The network of MF is assumed to be uniformly distributed in the CSK; therefore, the positioning radius and angles are drawn from uniform distributions (6).

6 $$\begin{aligned} \left\{ \begin{array}{l l} r_i={\mathscr {U}}\left( r_{\mathrm{NM}},r_{\mathrm{CM}}\right) \\ \theta _i={\mathscr {U}}\left( 0,2\pi \right) \\ \phi _i= {\mathscr {U}}\left( -\pi ,\pi \right) \end{array} \right. \end{aligned}$$

#### Microtubules

Unlike the two previous networks of filaments, microtubules (MTs) are able to sustain compression; therefore, they are rather considered as a network of interconnected springs. A particle of the set is assumed to interact with other particles of the set located at an initial distance lower than $l_{\mathrm{MT}}$. MTs are generated from the centrosome, namely a specific location in the CSK. Therefore, the network of MT is assumed to have a higher density in a preferential direction. This is reproduced drawing the two positioning angles from normal distributions centred on this specific direction, with a narrow specific variance $\sigma ^2_{\mathrm{MT}}=\frac{\pi }{2}$. The positioning radius, however, remains uniformly distributed (7).

7 $$\begin{aligned} \left\{ \begin{array}{l l} r_i={\mathscr {U}}\left( r_{\mathrm{NM}},r_{\mathrm{CM}}\right) \\ \theta _i={\mathscr {N}}\left( \mu =\theta _{CS}, \sigma ^2=\sigma ^2_{\mathrm{MT}} \right) \\ \phi _i= {\mathscr {N}}\left( \mu =\phi _{\mathrm{CS}}, \sigma ^2=\sigma ^2_{\mathrm{MT}} \right) ; \quad -\pi< \phi _i < \pi \end{array} \right. \end{aligned}$$

where $(\theta _{\mathrm{CS}},\phi _{\mathrm{CS}})$ is the direction of the centrosome, which is in turn drawn from uniform distribution due to its randomness (8).

8 $$\begin{aligned} \left\{ \begin{array}{l l} \theta _{\mathrm{CS}}={\mathscr {U}}\left( 0,2\pi \right) \\ \phi _{\mathrm{CS}}= {\mathscr {U}}\left( -\pi ,\pi \right) \end{array} \right. \end{aligned}$$

#### Focal adhesions

A fixed amount of focal adhesion (FA) is set initially. FAs are considered as static structures, part of the CM. Indeed, each FA is considered as a single particle, randomly disposed on the ventral part of the CM. As the number of FA does not evolve and their position is imposed, the dynamics of adhesion are not reproduced in the present model. FA particles are linked by pre-strained cables of stiffness $k_{\mathrm{FA}}$ and a positive pre-strain $\tau _{\mathrm{CM}}$ to the neighbouring particles of the CM at an initial distance $l_{\mathrm{FA}}$. Particles are uniformly distributed on the ventral part of the CM.

**Table 1:** Stiffness and pre-train values of the cables and spring intracellular interactions

	MT	IF	MF	SF
*k* (nN)	228 (Gittes et al. 1993)	157 (Gittes et al. 1993; Kojima et al. 1994)	10*	45.7 (Deguchi et al. 2006)
$\tau$	–	$-0.1$*	0.02*	0.2 (Deguchi et al. 2006)
	NM	CM	FA	CI
*k* (nN)	10*	10**	20	500*
$\tau$	–	0.02*	0.02*	–

#### Stress fibres

Stress fibres (SFs) are considered as longitudinal structures initiating from FA, and ending either on another FA or on the dorsal part of the CM. SFs are modelled as an alignment of particles. Particles are linked by cables of stiffness $k_{\mathrm{SF}}$ and positive pre-strain $\tau _{\mathrm{SF}}$ with the previous and the next particle of the alignment. The positive pre-strain induces an initial tension force in the SF, which reproduces their contractile behaviour induced by the action of myosin.

SFs are only considered when the cell is adhering on the substrate, namely when the cell is fully spread. When present in the model, SF are immediately pre-strained to account for contractility and, in the following, are able to be largely stretched or shortened due to external forces.

The generation of the SF is proceeded iteratively as follows: (i) starting either from the FA associated with the fibre or from the last generated particle of the SF; (ii) a cone is defined, which tip is located at the particle centre, vertical axis pointing towards the nucleus centre, height $\delta h_{\mathrm{max}}$ and opening angle $\alpha _{\mathrm{max}}$; (iii) the next particle is randomly positioned inside the cone; and (iv) the generation of the fibre stops if the newly positioned particle is located at a distance lower than $l_{\mathrm{SF}}$ from the dorsal part of the CM, or from another FA. Such generation process allows to form initially tortuous and non-uniformly distributed SF that interact through contact with the other intracellular structures of the CSK and the membranes.

#### Cytoskeleton interconnections

The IF, MF, MT networks are considered interconnected. A particle of any these sets is assumed to interact with a particle of any of the two other sets located at an initial distance lower than $l_{\mathrm{CI}}$. Identically, SFs are considered connected to the IF network. These connections are ensured using cables of stiffness $k_{\mathrm{CI}}$. In this way, the indirect connection between the actin CSK and MT with the NM through the IF observed in Green et al. (1986) can be reproduced.

#### Substrate

Finally, the substrate, either concave, convex or flat, is considered as perfectly rigid. The substrate is modelled as a rigid cluster of touching particles, which makes it perfectly impermeable. The substrate interacts only through contact with the CM and the FA.

### Adhesion simulation proceeding

In order to be able to compare strictly the influence of the substrate topography, cells are generated in an identical initial spherical configuration (Fig. 2a).

Simulations are conducted in two consecutive phases. The first phase consists in spreading the cell on the substrate. The spreading of the cell is achieved by pulling on the FA with an imposed displacement until reaching a given total resultant force at the FA $F_{\mathrm{sprd}}$. This force is computed as the sum of the magnitudes of the forces at the FA. The simulated spreading of the cell is not supposed to reproduce a specific spreading mechanobiological process, and the only purpose is to reach an adherent cell configuration from the identical initial configuration, in order to compare the adherent mechanical states from one configuration to another one. For comparisons of cells adhering on different substrates, an identical total resultant force at the FA $F_{\mathrm{sprd}}$ is chosen independently of the curvature. This choice is based on the hypothesis that cells tend to systematically apply similar forces on the substrate rather than conserving a similar spreading area (Sheetz et al. 1998).

The final configuration of the phase 1 is considered as the reference configuration of the phase 2. The spread state is considered as the initial state of all the strained mechanical structures of the cell, namely cables and springs. Therefore, all the internal forces in the cables and springs vanish, and the cell remains in its spread configuration, as it becomes its reference state. Starting from this configuration, the phase 2 begins. While in the phase 1, the motion of the cell was driven by pulling on the FA, in the phase 2, the only active mechanism is the activation and contraction of actin/myosin complexes. The positively pre-strained cables constituting SF, MF and the CM generate forces throughout the intracellular structure leading to the stabilization of the cell on the substrate (Fig. 2b).

Computation of mechanical state of the cell model is computed using a time discretization scheme with 2000 time-steps (approximately 1000 in each phase) of 0.1 ms each.

### Identification, calibration and verification of the model

The identification of the geometrical and mechanical parameters is relies on experimental properties found in the literature. Then, the calibration and the verification of the model rely on experimental results of nano-indented cells adhering on flat substrate, made available in Fang and Lai (2016). It is shown that the present model of adherent cell is able to reproduce the mechanical behaviour of nano-indented cells while applying realistic forces at the FA for a given spreading area.

#### Geometrical and mechanical identification

In its initial configuration, the cell is assumed to have a spherical shape of $20\,\upmu$m diameter and the cell nucleus is a sphere of $10\,\upmu$m diameter. In order to have sufficient degrees of freedom while limiting computational costs, spherical particles have a 1$\,\upmu$m diameter. The substrate is assumed to be larger than the spread cell in order to avoid boundary effects. The density of particles in the diameter is 1 particle per $\upmu \text {m}^2$. A total amount of 30 FA is considered throughout each simulation. Thus, the amount of SF in the model is 30 or a few less if some SF end on a FA.

Cables and springs constituting intracellular components stiffness and pre-strain values are referred in Table 1.

In Table 1, parameters marked with an * lack of experimental data from the literature; therefore, values are taken from previous work realized with a similar modelling technique (Milan et al. 2007, 2013). However, the CM stiffness $k_{\mathrm{CM}}$ (**) will be adjusted independently.

Remaining parameters of the model, namely particles density of each intracellular structure and interaction lengths, are arbitrarily chosen to fit best experimental results of macroscopic cell tests. The chosen densities of particles lead to a total of 15290 particles in the whole model, which repartition for each structure is recapped in Table 2.

**Table 2:** Number of particles for the description of each intracellular structure

	MT	IF	MF	SF	NM
Number	500	200	500	838	648

	NS	CM	CTPL	FA	
Number	377	8059	4138	30	

The chosen interaction lengths are recapped in Table 3, leading to a total of 118, 556 cables and springs interaction, as well as 49687 contact interactions in the spread configuration.

**Table 3:** Interaction lengths of cables and springs intracellular interactions

	$l_{\mathrm{MT}}$	$l_{\mathrm{IF}}$	$l_{\mathrm{MF}}$	$l_{\mathrm{SF}}$
Length ($\upmu$m)	10	10	10	$(h_{\mathrm{max}}=)5$

	$l_{\mathrm{NM}}$	$l_{\mathrm{CM}}$	$l_{\mathrm{FA}}$	$l_{\mathrm{CI}}$
Length ($\upmu$m)	5	2	5	0.1

#### Calibration

**Fig. 3:**
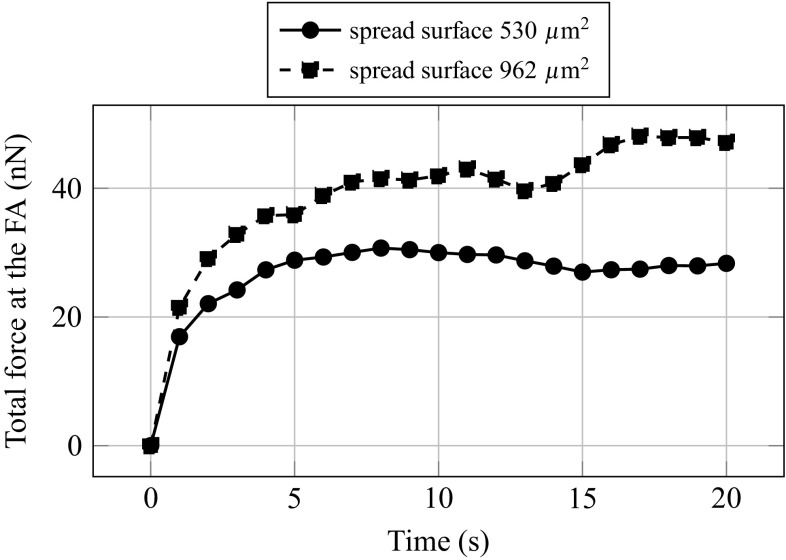
Total force at the FA evolution with time during the second phase of spreading when contractile forces are applied

The calibration of the model is achieved in three steps. First, the parameters are set following values presented in Table 3. Second, the spreading of the cell on a flat substrate is simulated, and all stiffnesses are proportionally adjusted to fit (Fang and Lai 2016) results in terms of total FA traction forces. By multiplying all stiffnesses from Table 3 by a calibration factor of 0.2, we are able to reach a total resultant force at the FA of $F_{\mathrm{sprd}} = 47.15$ nN for an adhesion surface of 962 $\upmu \text {m}^2$ (Fig. 3). Third, the nano-indentation test as proceeded in Fang and Lai (2016) is simulated and the stiffness of the CM $k_{\mathrm{CM}}$ is finally set in order to fit the final force of the test.

#### Verification

**Fig. 4:**
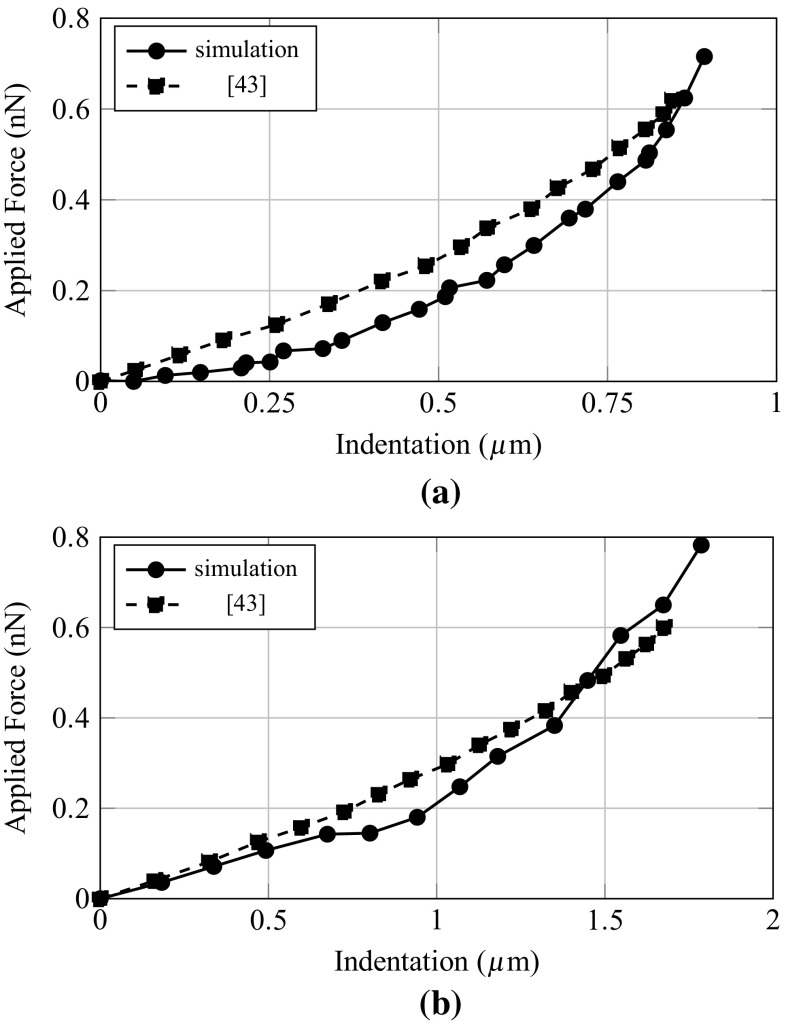
Force–displacement response of the cell model to a nano-indentation test **a** spread surface 962 $\upmu \text {m}^2$ **b** spread surface 530 $\upmu \text {m}^2$

The verification is achieved in two steps. First, we compare the reference and simulated nano-indentation curves obtained for a spread surface of $926\,\upmu \text {m}^2$ (Fig. 4a). In addition to the fact that the model could fit quite quantitatively reference results, we can observe that both curves have similar evolution, namely a parabolic trend. We can then assume that the model is able to reproduce the macroscopic mechanical behaviour of the reference cell, although it should be noticed that the cell model is too compliant at low indentation levels.

Second, the verification is pursued comparing the cell model—without modifying parameters—with reference results obtained for a spread surface of $530\,\upmu \text {m}^2$. The total resultant force at the FA at the end of spreading is $F_{\mathrm{sprd}} = 28.32$ nN (Fig. 3), and this second verification value is relatively close to the reference result 25.54 nN (Fang and Lai 2016). Results of the second nano-indentation test (Fig. 4b) are also quite similar to experiments, higher compliance of the cell when less spread, hence less strained, is observed. These results of the model demonstrate its aptitude to reproduce the macroscopic mechanical behaviour of adherent MSC at different spreading states.

We now consider that the implemented cell model is quite representative of the mechanical behaviour of MSC; we will use the reproducibility of this virtual cell to experiment on the influence of topography and of the constituents of the CSK on the mechanical state of the cell.

## Results

### Influence of topography curvature

**Fig. 5:**
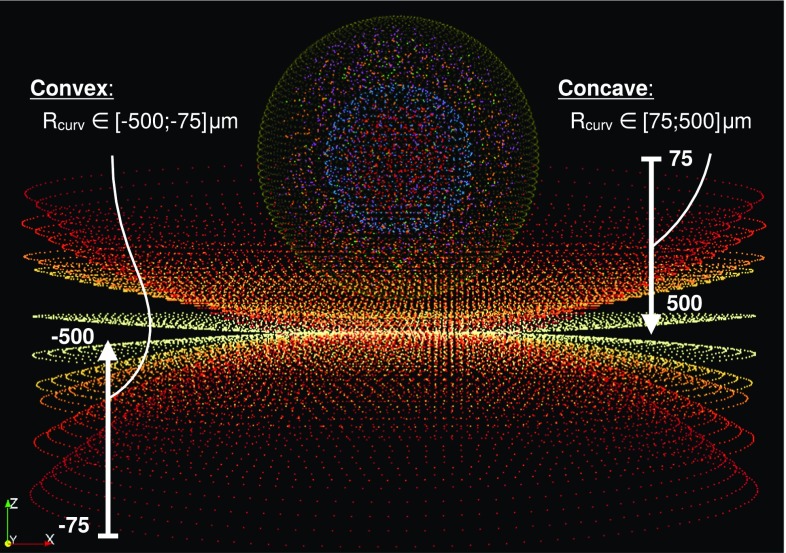
Visualization of the cell model and of the tested substrate topographies

In a first series of simulations, 10 identical cells are generated in a spherical state including all aforementioned intracellular constituents. The cells are spread on 10 different substrates. Substrates are cut hemispherical surfaces, either convex or concave with 10 different curvature radii $R_{\mathrm{curv}}$: $\pm 75$, $\pm 100$, $\pm 150$, $\pm 200$ and $\pm 500\,\upmu$m (Fig. 5). For convention, convex substrates are considered to have negative curvature radii. The 10 adherent cells are spread until the reach a given, identical for each cell, total force at the FA. Presented quantitative results in Figs. 7, 9, 10, 11 and 12 are averaged results on 5 random draw of the CSK of each configuration at each curvature radii.

*Qualitative observations*

**Fig. 6:**
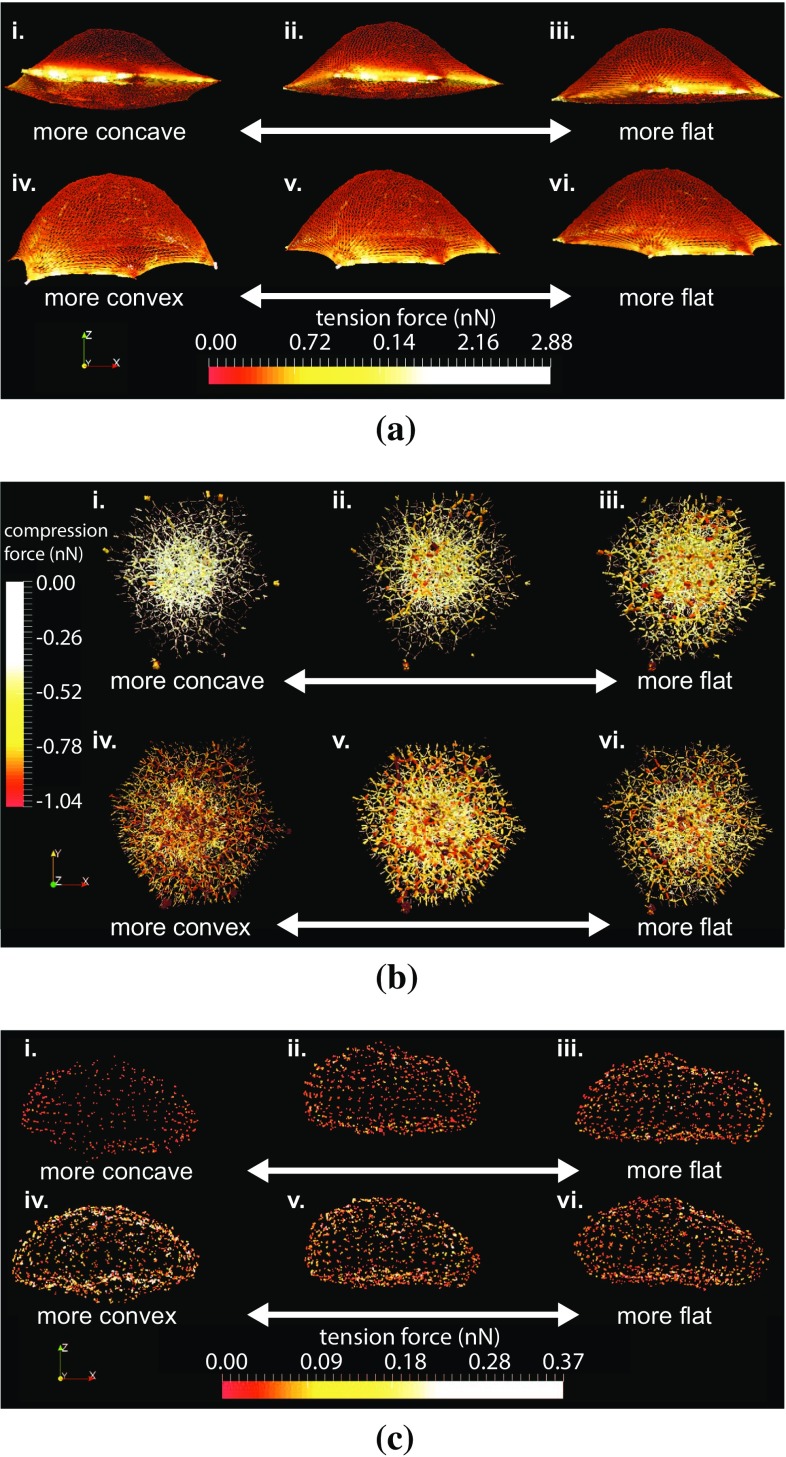
Influence of the curvature of the substrate on forces network (in nN) in the cell model. Concave topographies are located at the *top*, with increasing absolute curvature radius from left to right: (*i*) $75 \,\upmu$m, (*ii*) $200 \,\upmu$m and (*iii*) $500 \,\upmu$m. Convex topographies are located at the *bottom*, with increasing absolute curvature radius from left to right: (*iv*) $75 \,\upmu$m, (*v*) $200 \,\upmu$m and (*vi*) $500 \,\upmu$m. *Coloured spots* represent an interaction between two particles of the depicted structure: **a** the cell membrane, **b** the cytoskeleton and **c** the nucleus membrane. The tension force ranges from negative values in *red* associated with compression stresses, to positive values in *yellow* associated with tension stresses

Tension forces amplitude in the CM does not vary significantly from convex to concave topographies (Fig. 6a). Maximum tension forces are found in the farther parts of the CM from the cell centre. However, the more convex the topography, the more tensed the dorsal part of the CM. Compression forces in the CS and in the MT increase with the degree of convexity of the substrate (Fig. 6b), in terms of amplitude as well as of density. Tension forces in the NM increase as well with the degree of convexity of the substrate (Fig. 6c). More tension in the NM implies a more compressed NS. This observed mechanical state of the NM and of the NS can be explained by a greater vertical pressure applied on the nucleus by the CS, the MT and the CM, when the degree of convexity of the substrate is higher. This is consistent with observations made on the tension forces in the CM as well as on the compression forces in the CS and the MT.

*Quantitative observations*

**Fig. 7:**
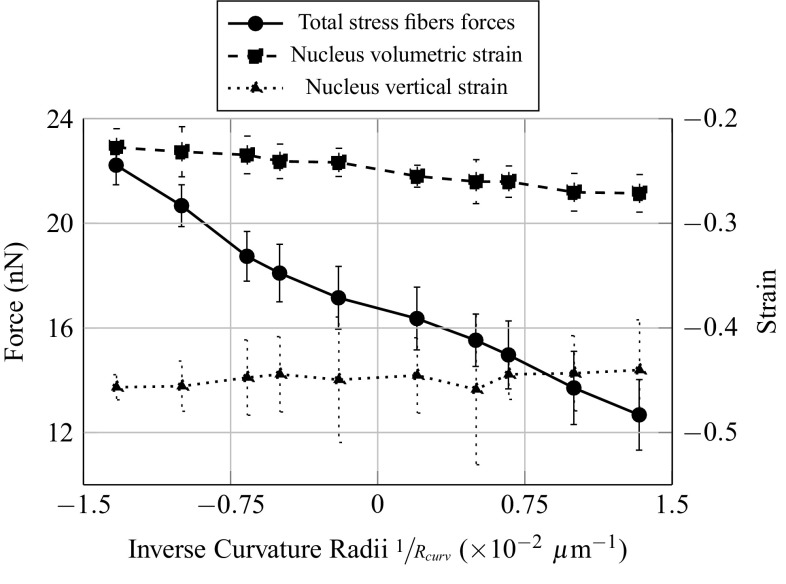
Influence of the curvature of the substrate on the total forces in the stress fibres, and the volumetric and vertical strains of the nucleus

Evolutions of forces and strains are plot as function of the inverse of the curvature radii, in order to have a continuous transition from more convex topographies in negative abscissa, to more concave topographies in positive abscissa. The total resultant force in the SF (Fig. 7), namely the sum of the magnitudes of the tension force in each of the SF, increases with the degree of convexity of the substrate, even though the force applied on the FA is identical on each substrate topography. A possible explanation could come from the fact that the shape of the SF varies from straight to curved, when the substrate topography varies from concave to convex. The compressive volumetric strain of the nucleus (Fig. 7) decreases with the degree of convexity of the substrate. This implies a more confined nucleus, or in other words higher hydrostatic pressure on the nucleus, for more concave substrate topographies. The compressive vertical strain of the nucleus (Fig. 7) seems to increase with the degree of convexity of the substrate. However due to important fluctuation of the measure for two consecutive topographies curvature, this result might not be significant.

**Fig. 8:**
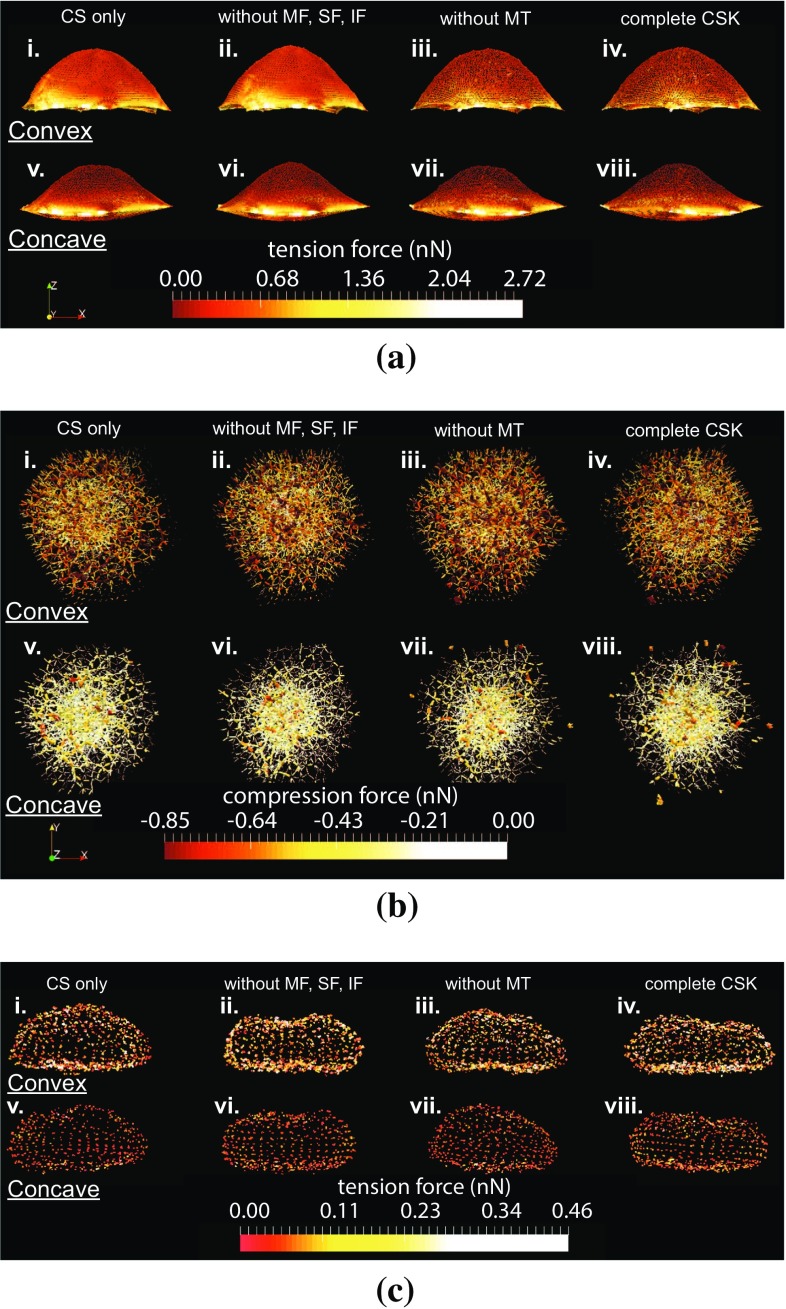
Influence of the CSK configuration on forces network (in nN) in the cell model. Convex substrate topographies of curvature radius $100 \,\upmu$m at the *top*, from left to right: (*i*) with CS only, (*ii*) without MF/IF/MT, (*iii*) without MT and (*iv*) with complete CSK. Concave substrate topographies of curvature radius $100 \,\upmu$m at the *bottom*, from left to right: (*v*) with CS only, (*vi*) without MF/IF/MT, (*vii*) without MT and (*viii*) with complete CSK. *Coloured spots* represent an interaction between two particles of the depicted structure: **a** the cell membrane, **b** the cytoskeleton and **c** the nucleus membrane. The tension force ranges from negative values in *red* associated with compression stresses, to positive values in *yellow* associated with tension stresses

**Fig. 9:**
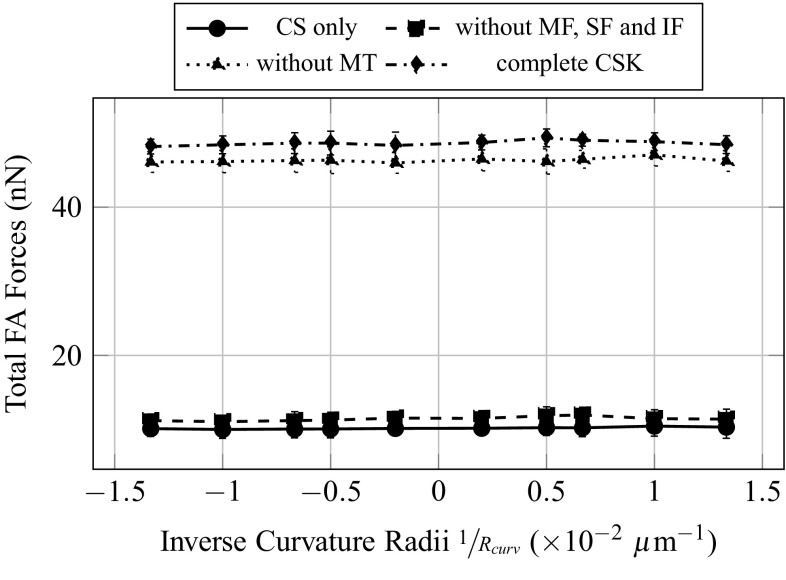
Influence of the curvature of the substrate on the total forces in the focal adhesions for different CSK configurations

**Fig. 10:**
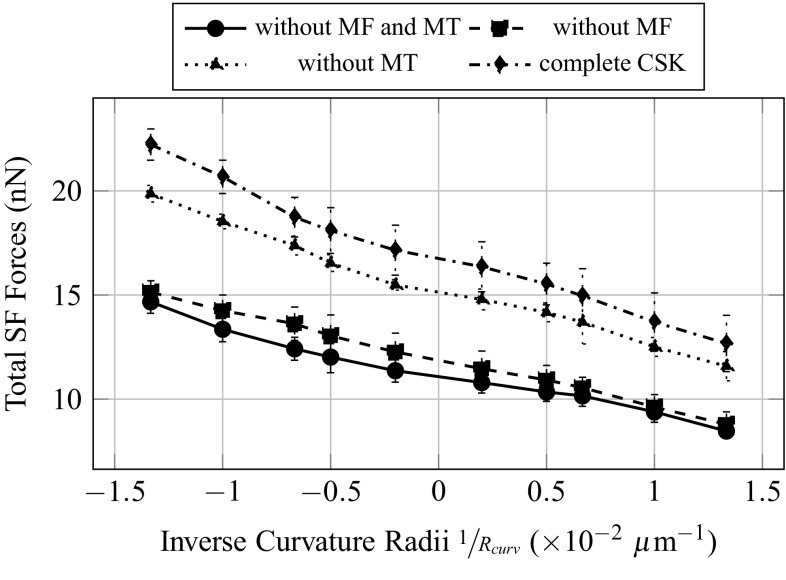
Influence of the curvature of the substrate on the total forces in the stress fibres for different CSK configurations

### Influence of intracellular structures

In a second series of simulations, the same analysis is realized for several configurations of the CSK: without MF, SF and IF; without MT; and with only CS. For qualitative observations, only one concave and one convex topographies are shown, respectively, with 100 and $-100$ $\upmu$m curvature radii.

*Qualitative observations*

Tension forces in the CM (Fig. 8a), either on concave or on convex substrate topographies, are less important in configurations when actin components of the CSK are considered. In addition, the shape of the dorsal part of the CM appears less inflated. Actin constituents of the inner CSK are pre-strained tensile structures as well as the CM, which incorporates the actomyosin cortex. Thus, they contribute in a similar fashion to the distribution of tension forces across the cell, hence the less tensed CM. Few differences can be observed in the compression forces network of the CSK (Fig. 8b), either on concave or on convex substrate topographies. Only in configurations without MT or with the complete CSK on convex topographies, we could tell that the compression forces appear slightly denser and higher. The CS and the MT are the only two constituents of the CSK able to sustain compression forces. Such weak differences in the compression forces network, whatever the CSK constituents considered, are mostly due to the fact that in absence of the MT, the CS will directly replace them without additional strain of the network. Indeed, the CS is here considered as an incompressible fluid. The shape of the NM (Fig. 8c) appears more squashed when MTs are introduced in the CSK. In addition, the tension forces in the NM seem to increase when more constituents of the CSK are introduced, independently of actin filaments or MT. Nevertheless, this effect is less visible on concave topographies. Finally, the presence or absence of an anisotropic distribution of MT did not alter the polarization of the other intracellular structures, and in particular the nucleus. Those structures remained relatively isotropic.

**Fig. 11:**
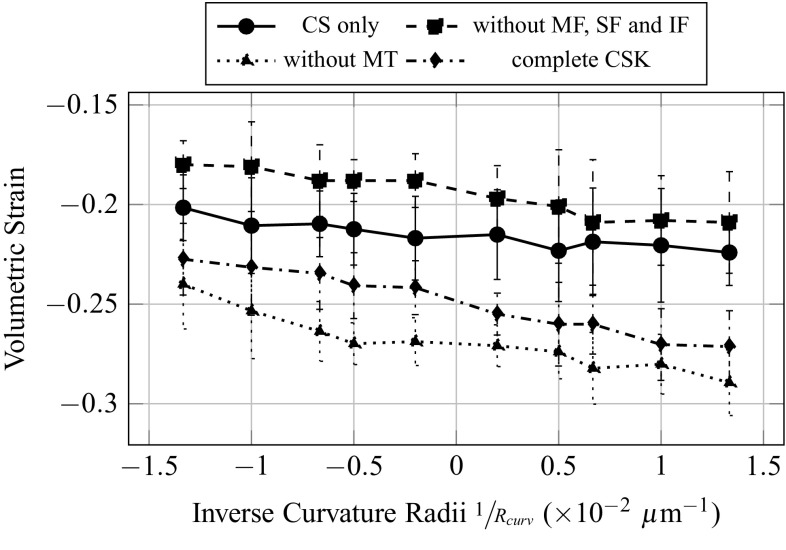
Influence of the curvature of the substrate on volumetric strain of the nucleus for different CSK configurations

**Fig. 12:**
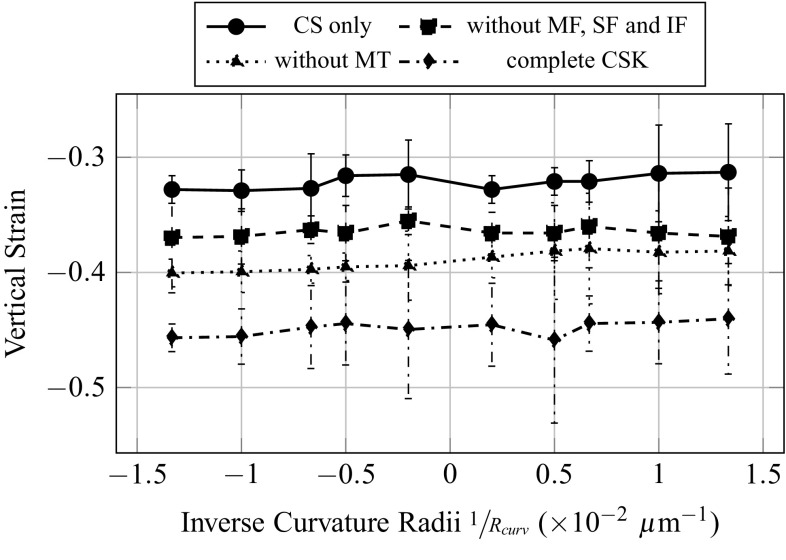
Influence of the curvature of the substrate on vertical strain of the nucleus for different CSK configurations

*Quantitative observations*

The total forces at the FA (Fig. 9) are greatly influenced by the addition of actin filaments in the inner CSK, and its value is four times as big when MF, SF and IF are considered, independently of the presence of MT. This increase in total forces at the FA is simply due to the contractile behaviour of the MF and SF. As expected from the calibration method, the total force at the FA does not vary with the curvature. The total forces in the SF (Fig. 10) are primarily dependent on the introduction of contractile actin in the inner CSK, namely MF, whatever the substrate topography. The total forces in the SF increase significantly, in average of 5nN with the first introduction of MF. This effect is even amplified with the second addition of the MT, an increase of 7.5nN, in average, is then observed. The addition of MT stiffens the CSK; therefore, applying a given pre-strain would lead to higher forces in the presence of MT. However, it is interesting to note that when the MTs are first and solely considered in the CSK, almost no increase in the total forces in the SF is noted. Opposite effects are observed on the compressive volumetric strain of the nucleus (Fig. 11), with the consideration of either the actin filaments or the MT. In the sole presence of the MT, the volumetric strain of the nucleus decreases, compared to the configuration where only the CS is considered, in average of 2.5%, which is equivalent to a decrease in the hydrostatic pressure or confinement on it. Inversely, in the sole presence of the MF, SF and IF, the volumetric strain increases in average of 5%. Unlike effects on the volumetric strain, cumulative, even amplifying effects are observed on the compressive vertical strain of the nucleus (Fig. 12). In the sole presence of MT, the compressive vertical strain increases, in average of 5%. In the sole presence of the MF, SF and IF, the vertical strain increases as well, even more significantly, in average of 7%. When the complete CSK is considered, then a cumulative effect is observed with an increase in the vertical strain in average of 10%.

## Discussions

From the simulations on several degrees of convexity of substrate, one can retain the following informations regarding the influence of the curvature on the mechanosensitive intracellular structures:

- The nucleus is less hydrostatically confined for higher degrees of convexity of the substrate, which makes it easier to deform. Thus, mostly from qualitative results, the NM appears more tensed laterally, in other words more stretched. This should coincide with a more compressed vertically nucleus; however, results do not allow to conclude significantly. Concave topographies seem to reduce vertical straining of the nucleus thanks to a confinement provided by the less important but more homogeneously distributed compressive stresses in the CS, around the nucleus. Higher hydrostatic pressure on the nucleus, in addition to increasing its stability, has been shown to drive mitotic cell rounding (Stewart et al. 2011). Therefore, concave topographies could be more suited for cell growth as observed in (Zinger et al. 2004).
- The SFs are more tensed on higher convexity substrates, which is unexpected. Forces in SF are considered null during the spreading phase. Forces only appear when pre-strain is applied, which is done identically in all simulations at 20%. This is supposed to reproduce the building of SF during adhesion and the constant pre-strained observed experimentally. Therefore, the tension in SF should be identical in every case. Higher tension on convex topographies might then be explained by geometrical constraints, namely the curved shape of the SF and the vertical reaction force applied by the substrate which prevent them from relaxing through straightening. Higher traction forces in the SF induced by convex curvature lead to global stiffening of the cell, based on the tensegrity modelling of the CSK. It has been shown to avoid early apoptosis (Wang et al. 1993; Pelling et al. 2009) and might further lead to cell growth and division. Furthermore, when considering the vectorial resultant force of the tension forces in the SF, the convexity of the substrate is even more significant. Indeed, in the case of a concave substrate, the SFs are almost straight and contained in the horizontal plane; therefore, the vectorial sum is zero. However, in the case of a convex substrate, the SFs are bent; therefore, the vectorial resultant force is vertical pointing downwards, pushing on the nucleus.
- The CM is more tensed on higher convexity substrates, in particular when comparing the dorsal CM for different curvatures. Stretching on the CM is known to have a direct influence the activation of ion channels (Gustin et al. 1988). Inversely, a decrease in tension in the CM facilitates trafficking events requiring membrane flexibility (Dai et al. 1997; McMahon and Gallop 2005). Diverse signalling events can be expected from these stretch increase or decrease and thus have various consequences on cell fate (Martinac 2004; Farge et al. 1999).

The orientation and position of the nucleus are critical during mitosis because it determines the cell division axis (Théry et al. 2005). Thus, the stability of the adherent cell and of its nucleus is predominant to control the cell fate. We have shown that intracellular forces increase as a whole with the degree of convexity of the substrate, leading to an increase in the stiffness of the cell. This contributes to a partial increase in stability. However, this effect is counterbalanced by the simple fact that concave topographies are stable mechanical equilibrium configurations, being minima of potential energy, while convex are unstable ones. In an unstable equilibrium, a slight mechanical perturbation of the cell could lead to significant motion and rearrangements. The competition between these two effects should be further investigated, but at a first glance and considering observation on the rounding-up of the cell, concave topographies seem favourable for regular cell division.

If the choice of migration towards concave did not rely solely on haptotaxis (Kim et al. 2012), but also on preferences of the cell for more stable configuration favourable for division, the probing of the environment relying on the lamellipodium would be primordial. This could explain why cells tend to less prefer concave when Arp2/3 is inhibited (Song et al. 2015).

The testing of different configurations of the CSK helped to analyse the role of the actin and MT networks during the spreading and the adhesion of the cell:

- The actin network constituted of SF and MF increases tension in the cell and forces at the FA due to contractile properties. The actin network therefore helps the spreading of the cell as well as of the nucleus.
- From the influence of MT on the NM shape, the CM shape and the nucleus strains, it can be inferred that the MTs shield the nucleus from hydrostatic as well as uniaxial stresses by sustaining CM and CSK.
- The MT increases tension in the structure, but only if the actin network is present, which is consistent with tensegrity. The MTs constrain large structural changes in the actin cable network, since both are interconnected, increasing the tension in the actin network.

This analysis of the role of the actin and MT networks helped verifying the consistency of the results given by the model.

In the present simulations, the behaviour of the model was almost quasi-static. There was few dependence of the results to time, in particular because no mechanobiological process is considered, as well as no dissipative mechanisms. If phenomena dependent on mechanotransduction were to be considered, visco-elasticity would have been almost mandatory. Visco-elasticity would delay the triggering of mechanobiological processes, which are conditioned by the mechanical state of the intracellular structures, such as the nucleus. Since the vertical strain of the nucleus is linked to the vertical pressure applied by the cell membrane, visco-elasticity might have had some influence. In parallel, if cell membrane were visco-elastic, in the non-stationary state of the cell membrane, increased tension forces would have to be supported by the stress fibres, which could trigger additional mechanisms.

## Conclusion

This study has consisted in the development of an adherent cell model by means of discrete rigid bodies and tensegrity, considering explicitly mechanically relevant cells internal structures. The model has been calibrated using as much experimental data available in the literature as possible. Once final parameters were finally calibrated fitting the total FA forces for a given spreading area, the model has been verified comparing nano-indentation tests from the literature.

Influence of the curvature of the substrate topography, ranging from concave to convex, on the mechanical cell behaviour has been investigated. The comparison has been realized at identical total FA forces on each curvature. The dependency of nucleus vertical and volumetric strains, SF total forces, CM tension forces and CS compression forces to the curvature and to the CSK constituents considered has been analysed. Relations between curvature and mechanical state of intracellular structures, and possible consequences on their relative mechanosensitivity have been proposed. A specific attention has been brought to the nucleus mechanical state dependence on the curvature, and its possible consequences on cell division and gene expression.

The present model helped to discriminate the influence of the topography in particular for the nucleus. However, the analysis is limited to the macroscopic mechanical state of the nucleus. Because of the significance of the nucleus with respect to many mechanobiological events, further and more detailed attention must be paid to the consequences of the mechanical state of the nucleus on its constituents (Swift et al. 2013), in particular chromatin which is known to regulate gene expression (Jong 2002; Rabineau et al. 2015). In addition to the consideration of multi-scale mechanical cues, dynamic mechanobiological processes will have to be integrated to the model for further investigations. Indeed, the reorganization of the CSK, by means of filaments polymerization and depolymerisation; the force regulation in components, such as the SF; and the focal adhesion dynamics condition significantly the final mechanical state of the cell. In future attempts to model cell adhesion on elastic fibrous substrates, cell force regulation in response to substrate stiffness variations will have to be specifically addressed (Trichet et al. 2012; Crow et al. 2012).
